# Perception of health and illness and quality of life after total thyroidectomy for differentiated thyroid carcinoma: the PERSAM study

**DOI:** 10.3389/fendo.2024.1472448

**Published:** 2024-12-26

**Authors:** Elena Groff, Beatrice Soccal, Fabiola Carrossa, Federica Vianello, Sara Talomo, Alessandra Feltrin, Giovanni Scarzello, Honoria Ocagli, Dario Gregori, Matteo Martinato

**Affiliations:** ^1^ Hospital Psychology, Veneto Institute of Oncology IOV-IRCCS, Padua, Italy; ^2^ Medical Oncology, Veneto Institute of Oncology IOV-IRCCS, Padua, Italy; ^3^ Radiotherapy, Veneto Institute of Oncology IOV-IRCCS, Padua, Italy; ^4^ Unit of Biostatistics, Epidemiology and Public Health, Department of Cardiac-Thoracic-Vascular Sciences and Public Health, University of Padova, Padua, Italy

**Keywords:** quality of life, perception of health and illness, thyroidectomy, differentiated thyroid cancer, radioactive iodine therapy

## Abstract

**Background:**

Differentiated thyroid carcinoma is the most common endocrine neoplasm; several studies have shown that individuals perceive the disease as being more severe than it actually is, resulting in a reduced quality of life. The primary aim of this study is to assess the quality of life and perception of illness among patients admitted for radiometabolic therapy, post total thyroidectomy for differentiated thyroid carcinoma. The secondary aim is to identify which patient characteristics are associated with a lower quality of life in order to improve and personalize care.

**Methods:**

The study was conducted at the UOC of Radiotherapy Veneto Institute of Oncology IOV-IRCCS in Padua, Italy. Three questionnaires were administered: Psychological General Well-Being Index (PGWBI), the Illness Perception Questionnaire (IPQ-R) and the Short Form Survey (SF-12). A descriptive statistics analysis and multiple linear regression models were performed to explore the relationship between some of the variables.

**Results:**

Significant associations emerged between the type of surgery and higher values on the PGWBI questionnaire (P = 0.022) and the score obtained in the ‘emotional representations’ dimension of the IPQ-R questionnaire (P = 0.028). Pathology staging was statistically significantly (P = 0.026) associated with the score obtained in the dimension ‘identity’; age with the scores obtained in the dimensions ‘emotional representations’ (P = 0.035), ‘personal control’ (P = 0.004), ‘consistency with pathology’ (P < 0.001) and ‘causes’ (P = 0.004).

**Conclusions:**

There is evidence of moderate distress in psychological well-being and good perception of pathology. There is less understanding of the disease in individuals with stage 3 and 4 thyroid cancer, in those who have undergone total thyroidectomy without lymphadenectomy and who are aged over 60. Physical well-being: limitations in self-care and moderately physically demanding activities. Mental health: more information to patients decreases the degree of stress and promotes ‘positive’ emotions. Physical health status: a long-term activity program, characterized by aerobic exercises to be performed in groups or at home, is useful. This study allows to transpose the results into clinical practice, evaluating the possibility and methods of providing personalized care to patients.

## 1 Introduction

Differentiated thyroid cancer (DTC) is the most common endocrine neoplasm in the world.

Sexual dimorphism has an impact on cancer biology, with most cancers being male-dominated. Thyroid cancer is a non-reproductive cancer and has a clear female predominance, with an incidence three to four times higher among females, although males generally have a more aggressive disease. To date, however, there is no known molecular evidence for this. Consequently, current approaches to treatment and surveillance are not gender-specific (1).

Instead, other authors argue that different disease frequencies may depend on differences in patient behavior and thyroid investigations. Sex hormones may be influential, but this is something that has not yet been clarified. Estrogen may increase the production of mutagenic molecules in the thyroid cell and promote the proliferation and invasion of tumor cells by regulating both the enzymatic mechanism of the thyrocyte and the inflammatory process associated with tumor growth. In addition, there is a lack of literature regarding the worse prognosis of thyroid cancer associated with male sex (2).

Although it is characterized by a favorable prognosis and a low incidence of recurrence, the perception of health and quality of life, which depend on the type of carcinoma, may vary: individuals may perceive their disease to be more severe than it actually is and have a significantly lower quality of life than the general population (3).

The study enrolled 110 patients (91 women) with an average age of 53.5 years, and the level of education was the only socio-demographic factor able to influence the patients’ perception of the disease. No correlation was identified between the patient’s perception of the disease and the stage of the disease. Among the disease severity parameters, time since last treatment, evidence of disease persistence and number of treatments were associated with a negative perception of the disease. The authors of the study concluded by stating that patients with DTC perceive their disease on a subjective and emotional basis not related to its actual severity. They also suggest that in order to improve patients’ representations of the disease and their level of quality of life, a qualified psychologist should be included in the multidisciplinary team managing these patients and that special attention should be paid to less educated patients and patients requiring repeated iodine treatments. The dimensions quality of life and level of psychological well-being were not studied in this study.

It was therefore planned to conduct this non-profit study with the aim of confirming or negating some of the results of the study described above in a different setting, with an assessment of the level of quality of life conducted by means of a more generic instrument and simultaneously measuring the psychological well-being of the patients under study.

For the purposes of this study, in order to exclude potential confounding factors, only patients undergoing radical surgical treatment followed by radio-metabolic therapy (ablative treatment with radioiodine 131I) and hormone therapy were considered. Patients in this study received iodine therapy between 6- and 12-weeks post-surgery. In order to assess the psychological areas of interest in the present study, three self-report psychological assessment questionnaires were identified that were capable of analyzing these constructs quickly and effectively.

## 2 Methods

In order to assess the psychological areas of interest in the present study, three self-report psychological assessment questionnaires were identified, validated for the population considered and capable of analyzing, from the patient’s perspective, the constructs of quality of life, perception of illness and psychological well-being. The study was conducted at the Radiotherapy Unit of the IRCCS -Veneto Institute of Oncology in Padua.

### 2.1 SF-12

The SF-12 (Short Form Survey) (4) is a questionnaire that measures the patient’s level of physical and mental health. It consists of 12 items that produce measures relating to two different aspects of health: physical health (PCS-12) and mental health (MCS-12). The SF-12 consists of four scales (physical functioning, role and physical health, role and emotional state, mental health) each measured by two items and four scales each measured by one item (physical pain, vitality, social activities and general health). Specifically, the 12 items describe the following dimensions: general health (GH), physical functioning (PF), role physical (RP), bodily pain (BP), vitality (VT), social functioning (SF), role emotional (RE) and mental health (MH). The scale scores are calculated by a standardized scoring algorithm that sums the responses between the scale items and then transforms the raw scores into a scale from 0 to 100 for each of the summary scores where 50 is the mean of the general population with a standard deviation of about 10. Higher values indicate a better perception of health (5). The questionnaire has been used in several quality of life studies specifically in the area of thyroid diseases, including DTC (5–9).

### 2.2 IPQ-R

The IPQ-R (Illness Perception Questionnaire) (10, 11) is a questionnaire that provides a complete picture of a patient’s mental representation of illness (perception of the state of health/illness). The questionnaire has also been used specifically in the field of thyroid diseases, including DTC (3). It consists of three sections:

- Illness identity: The items concern the patient’s beliefs about the nature of the illness. The section contains a list of 14 symptoms. The patient is asked to estimate whether or not they have experienced each symptom since the beginning of their illness by means of a “yes/no” answer. Then, adopting the same response system, the patient is asked to make a judgement of the relationship between the symptom and the illness. The first part of the questionnaire does not produce a score, but rather identifies the symptoms present, whether they are present at the onset of the illness or at the time of administration of the questionnaire.
- Opinions about the illness: the 38 items that make up this section describe how the patient perceives his or her illness. The patient rates the statements in the questionnaire using a five-point Likert scale: “strongly disagree”, “disagree”, “neither agree nor disagree”, “agree”, “strongly agree” (range 1-5).
- Causes of illness: eighteen possible causes of illness are listed, and the patient is asked to express, via the same Likert scale used in the previous part, his or her perception of the causes of the illness (range 1-5). Finally, the patient is asked to list, in order of importance, the three main causes of their illness, either by choosing from those listed or by indicating others of their own (12).

The questionnaire is available in the validated Italian version which was used without modification to conduct this study.

### 2.3 PGWBI

The PGWBI (Psychological General Well-Being Index) (13) is a questionnaire used to measure the subjective state of well-being or discomfort related to the emotional and affective sphere. The questionnaire consists of 22 items investigating six different dimensions: anxiety, depression, positivity and well-being, personal control, general health and vitality. The six scales consist of a minimum of three and a maximum of five items. The anxiety scale comprises five items, the well-being and vitality scales four items, and each of the remaining scales consists of three items. The score attributable to individual answers ranges between ‘0’ (the worst possible state) and ‘5’ (the best possible state). Depending on the number of items included in a specific dimension, the scale score ranges from a minimum of 0 to a maximum of 15-20-25 points. The PGWBI allows the calculation of an overall index, which can reach a theoretical maximum of 110 points (i.e. the unweighted sum of the responses to the individual items of all dimensions). A low score (0–60) on the PGWBI test indicates a high level of psychological distress; low values in this range are associated with poor quality of life, high anxiety, sadness and tension, while a medium score (61-72) indicates moderate psychological well-being, but with some presence of distress (this level suggests that the person may face stress or emotional challenges that partially affect well-being).

The questionnaire is available in the validated Italian version which was used without modification to conduct this study (14).

The scale has also been used in studies of patients with thyroid disease (15, 16).

### 2.4 Study design

The study, with an observational, cross-sectional, single-center, non-profit design, after being approved by the Area Nord Veneto Territorial Ethics Committee, was conducted at the Complex Operative Unit (UOC) of Radiotherapy IRCCS - Veneto Institute of Oncology in Padua. Validated questionnaires measuring quality of life and perceptions of health and illness were administered to patients admitted for radiometabolic therapy. The study was explained to them, and the purpose of the research described. All patients who gave their consent enrolled in the study. Consent to participate was voluntary and uncoerced.

### 2.5 Sample

The sample studied consisted of persons admitted as in-patients to the Radiotherapy Department - Protected Section. The inclusion criteria were:

- sex: male or female
- admission diagnosis: papillary carcinoma or follicular carcinoma stage: I, II, III or IV
- history of total thyroidectomy (with or without lymphadenectomy)
- treatment method, pre radiometabolic therapy, with thyroid-stimulating hormone (rhTSH)
- subjects undergoing radiometabolic treatment at the Radiotherapy unit of the IRCCS - Veneto Institute of Oncology in Padua.

Patients with poorly differentiated carcinoma and who did not meet the inclusion criteria listed above were excluded.

A power analysis was conducted to determine the appropriate sample size for the study to target the capability of the PGWBI to differentiate its levels across patient characteristics. The approach was developed to identify differences between strata (e.g., gender, age, and disease staging) in the patient populations with an effect size (ES) of at least 0.5. Based on a type-I error probability of 0.025 and a power of 0.90, the study requires 200 subjects overall (i.e., if strata are balanced, this is equivalent to 100 patients per stratum) to be enrolled.

### 2.6 Data analysis

The data obtained were organized in an Excel database, assigning an identification code to each patient to ensure anonymity. The statistical analysis performed was descriptive and delineated the continuous variables by means of first, second (median) and third quartile indices, while the categorical variables were delineated by means of percentages and frequencies. Wilcoxon’s nonparametric test was used for continuous variables, chi-squared for ordinal categorical variables, and Pearson’s test for unordered categorical variables. Multiple linear regression models of the PGWBI, IPQ-R and SF-12 questionnaire scores were used to meet the study objective. Statistical significance was considered for p-values below 0.05. For each regression analysis, two separate models where conducted to assess associations with the PGWBI and SF-12 (PCS-12 and MCS-12) scores. The first model included the full sample, encompassing all subjects. The second model, was focused only on subjects with at least one comorbidity. This division allowed to examine associations within the full population as well as within a higher-risk subgroup.

Kernel density estimation were used to visualize the distribution of PGWBI and SF-12 as a continuous curve that estimates the probability density of the data at each point, highlighting areas of higher and lower concentration while the area under the curve equals 1, allowing peaks to be interpreted as areas where the data are more concentrated. The overall shape of the score distribution allows identifying any deviations from normality. The bandwidth parameter is adjusted to achieve a smooth curve representation, accurately reflecting the observed data distribution

## 3 Results

271 questionnaires were collected. Of these, 21 patients who did not complete the forms administered, 19 individuals whose clinical records were not available for consultation and three participants whose diagnosis was poorly differentiated carcinoma were excluded. The characteristics of the sample of 228 patients are detailed in **Table 1**.

**Table 1:** Sample characteristics.

Variable	Method	N	%
Sex	Female	166	73%
	Male	62	27%
Comorbidities	Yes	105	46%
	No	123	54%
Number of comorbidities	1	56	53%
	2	29	28%
	3	18	17%
	4	1	1%
	5	1	1%
Type	Papillary carcinoma	201	88%
	Follicular carcinoma	27	12%
Stage	First	122	54%
	Second	84	37%
	Third	9	4%
	Fourth	13	6%
Intervention	Total thyroidectomy	223	98%
	Total thyroidectomy with lymphadenectomy	5	2%

### 3.1 Scoring of the PGWBI

**Figure 1:**
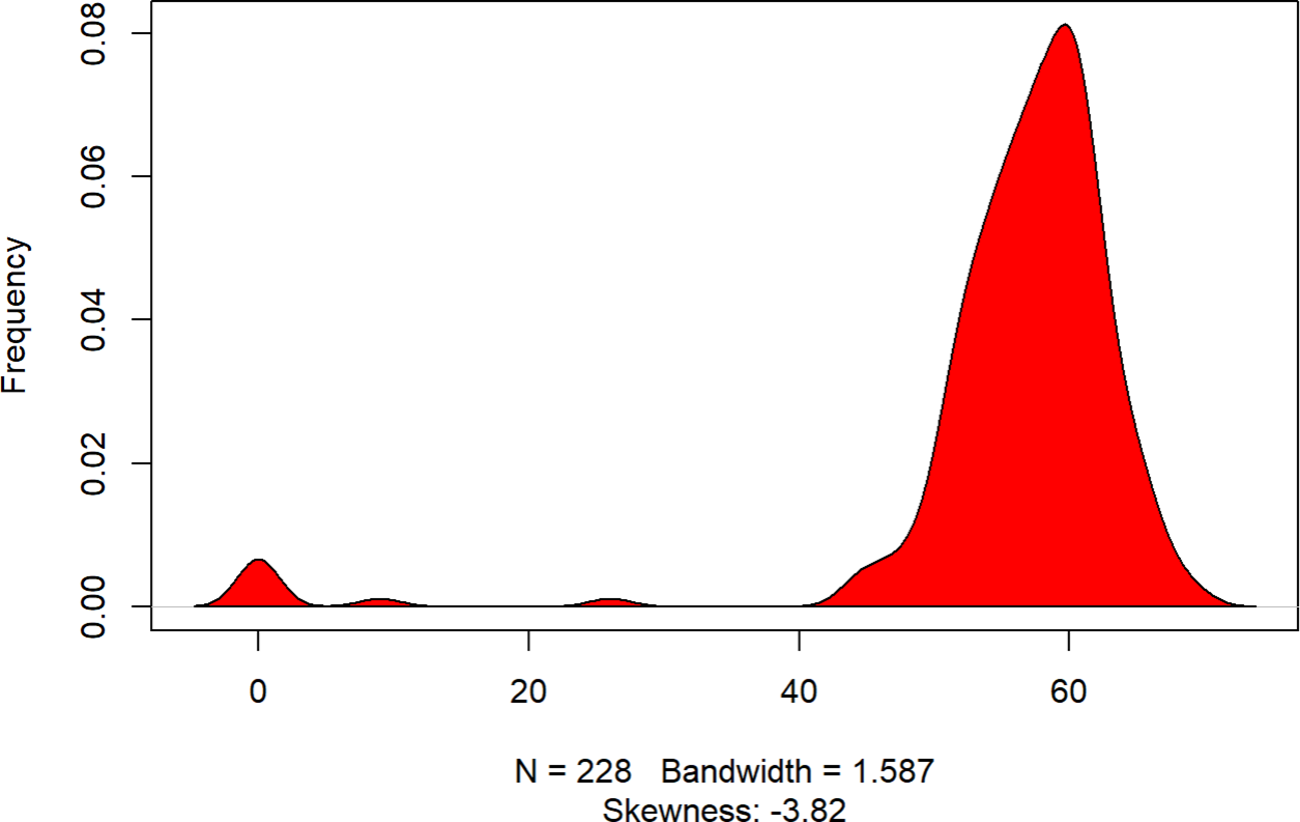
Density Plot: PGWBI score. The x-axis represents the range of PGWBI values, while the y-axis shows the estimated frequency of observations across this range. A significant skewness (-3.82) indicates a concentration of values at the higher end, suggesting that most participants have higher scores. The bandwidth parameter (1.587) controls the smoothness of the curve, ensuring a balance between detail and clarity in the visualization.

In relation to the scoring of the PGWBI questionnaire, scores below 40 were excluded as the distribution was unbalanced. **Figure 1** shows the data from the questionnaire. The patients presented a median score of 58 (Q1:54, Q3:61) in the dimensions ‘anxiety’, ‘depression’, ‘positivity and well-being’, ‘personal control’, ‘general health’ and ‘vitality’ out of a theoretical maximum of 110 points.

The kernel density plot (**Figure 1**) reveals a highly skewed distribution of PGWBI, with a majority of observations clustered towards higher values. This pronounced skewness (-3.82) suggests that most participants exhibit a high score (moderate psychological well-being), with only a few at lower levels.

### 3.2 Scoring of the IPQ-R questionnaire

The IPQ-R consists of 9 dimensions whose scores, according to the manual, cannot be added together, therefore the items were compared on the basis of carcinoma type, staging, intervention type and age. **Table 2** shows the overall results of the nine dimensions addressed by the questionnaire.

**Table 2:** Scoring of the IPQ-R questionnaire.

Variable	N	Statistics (I, II, III quartile)
Identity score	228	2.0/5.0/8.2
Timeline score	228	10/14/18
Consequences score	228	21/27/32
Personal control score	228	14/18/21
Treatment score	228	50/56/62
Illness score	228	14/18/20
Timeline cyclical score	228	6/8/11

The overall scores correspond to the three sections of the questionnaire.

Section 1 - Identity: the dimensions describe the patient’s beliefs about the nature of the illness. The sample obtained a median score of 5 (Q1:2, Q3:8.2) in a range from 0 to a theoretical maximum of 28 points.

Section 2 - Opinions about the illness: consisting of seven dimensions describing the subject’s considerations about the disease. The sample presented a median score of 14 (Q1:10, Q3:18) in the dimension ‘timeline’, a median of 27 (Q1:21, Q3:32) in the dimension ‘consequences’ and a score of 18 (Q1:14, Q3:21) in the dimension ‘personal control’ in a range from 6 to a theoretical maximum of 30 points. The sample then showed a median of 56 (Q1:50, Q3:62) in the dimension ‘treatment control’, a median score of 18 (Q1:14, Q3:20) in the dimension ‘coherence’ and a score of 18 (Q1:12, Q3:20) in ‘emotional representations’ in a range from 5 to a theoretical maximum of 25 points each. In the dimension ‘cyclicality’, patients expressed a median score of 8 (Q1:6.0, Q3:11.0) in a range from 4 to a theoretical maximum of 20 points.

Section 3 - Causes of the illness: the sample presented a median score of 41 (Q1:29, Q3:47) out of a theoretical maximum of 30 points.

### 3.3 Scoring of the SF-12 questionnaire

**Figure 2:**
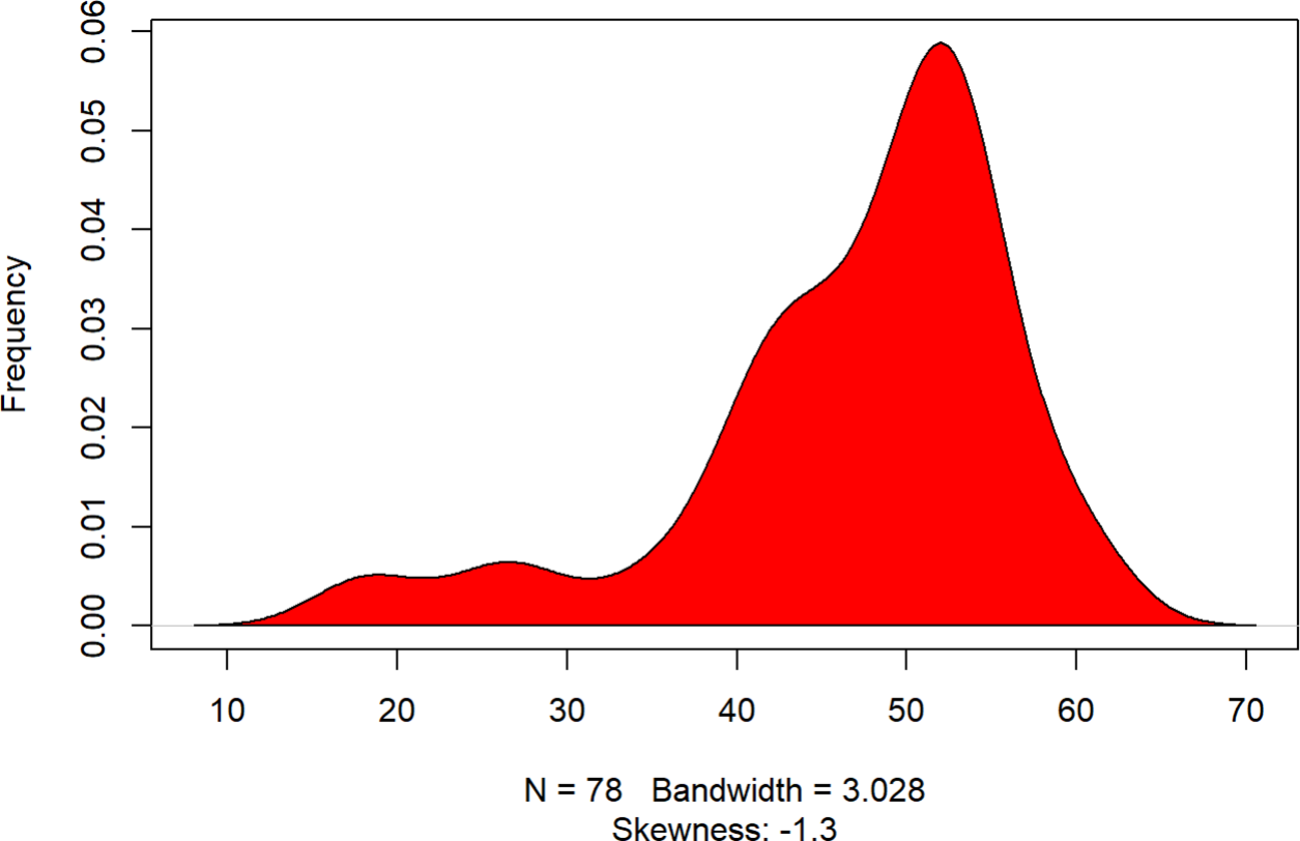
Density Plot: PCS12 score. The x-axis represents the range of observed values, and the y-axis represents the estimated frequency of observations within this range. The skewness statistic (-1.3) indicates the asymmetry of the distribution, with most values concentrated toward the higher end. Bandwidth (3.028) controls the smoothness of the curve to provide a clear visualization of the data distribution.

**Figure 3:**
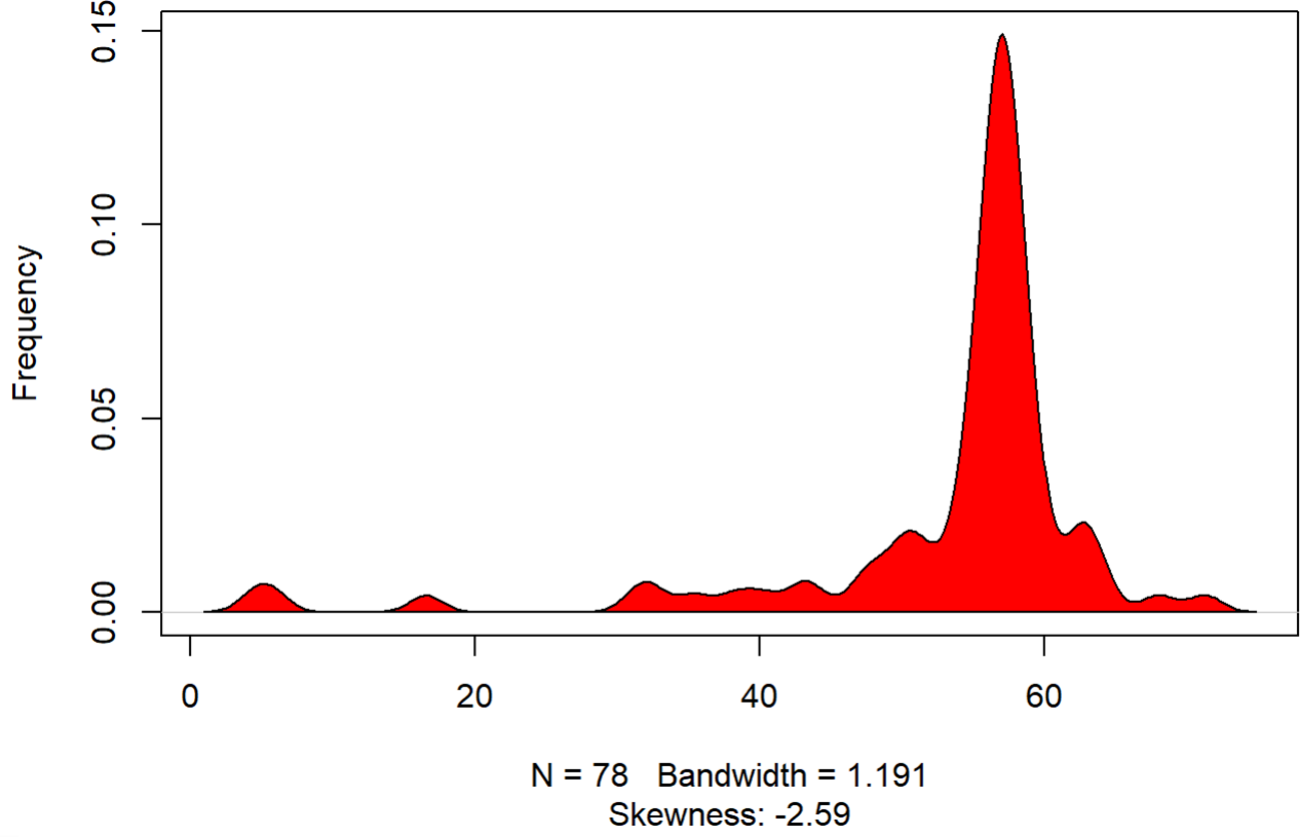
Density Plot: MCS12 score. The x-axis represents the range of observed values, and the y-axis represents the estimated frequency of observations within this range. The skewness statistic (-2.59) indicates the asymmetry of the distribution, with most values concentrated toward the higher end. Bandwidth (1.191) controls the smoothness of the curve to provide a clear visualization of the data distribution.

The PCS-12 and MCS-12 scores depicted in **Figures 2**, **3** represent an average and above-average health status of the study sample compared to the general population, respectively, while some subjects on the PCS-12 obtain lower scores (left-hand side of the plot between 15 and 35) indicating below-average physical or mental health. **Figure 2** represents the scoring of PCS12 (Physical Component Summary).

**Figure 3** below shows the MCS 12 (Mental Component Summary) score.

The patients obtained a median score of 57 (Q1:51, Q3:57) in the dimensions ‘vitality’, ‘social activities’, ‘emotional state’ and ‘mental health’ out of a theoretical maximum of 100 points.

### 3.4 Inferential statistical analysis

#### 3.4.1 Multiple linear regression model for the PGWBI questionnaire

The following variables were merged in the PGWBI questionnaire in order to present them in a less disproportionate manner: number of comorbidities (1 and > = 2) and staging (1, 2, 3-4).

**Figure 4:**
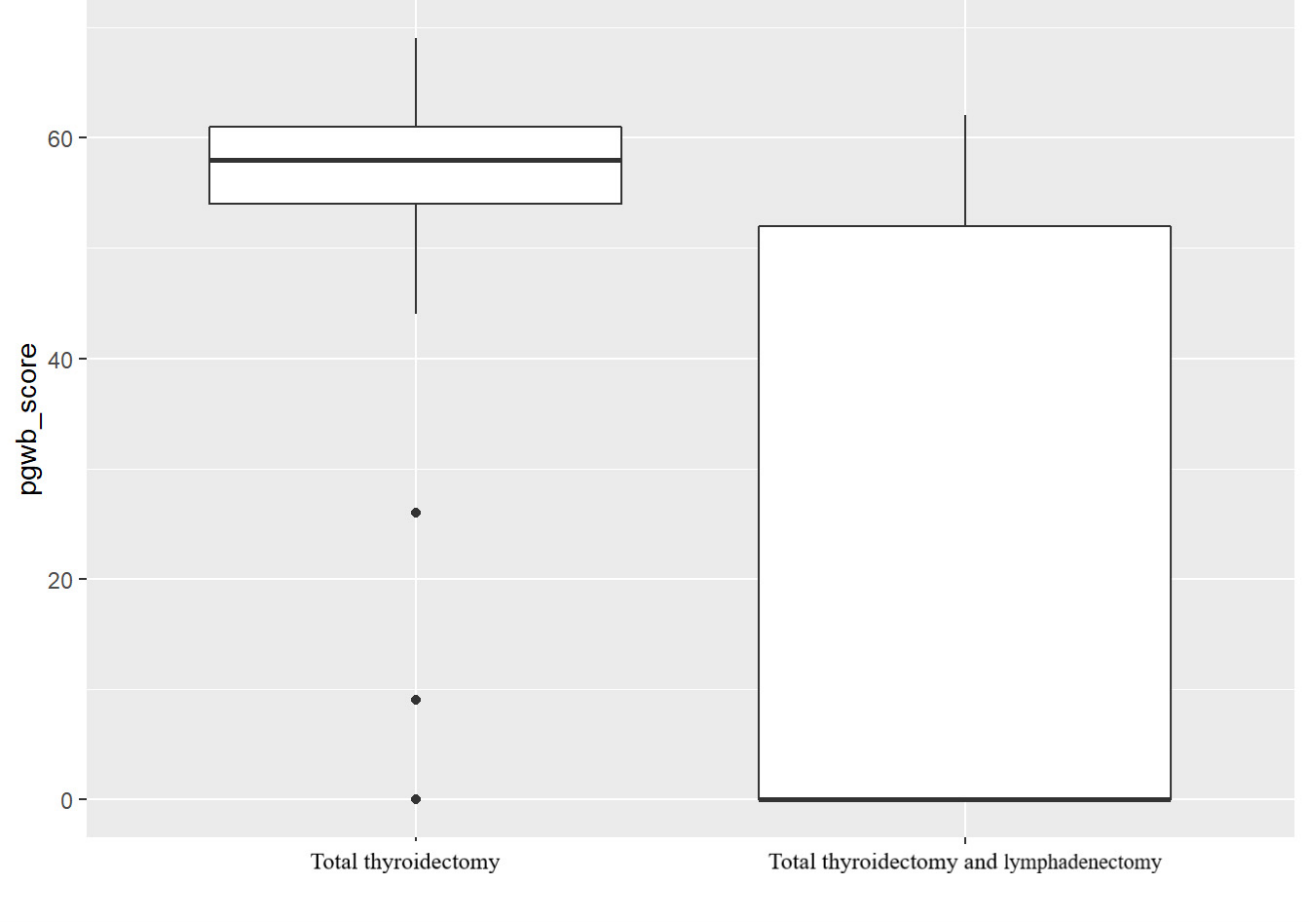
Box plot Multiple linear regression model PGWBI score.

The multiple linear regression model identified the type of intervention as statistically significant (P = 0.022) in relation to the subjective state of well-being or discomfort related to the emotional and affective sphere of the person (**Figure 4**).

Age (P = 0.489), sex (P = 0.356), carcinoma type (P = 0.675), disease staging (P = 0.453, P = 0.970) and number of comorbidities were not statistically significant neither in the whole sample (**Table 3**) and in the sample without comorbidities (**Supplementary Table S1**) in relation to quality of life.

**Table 3:** Multiple linear regression model for PGWBI questionnaire.

Variables	Estimate	CI	P-Value
Age	-0.02	-0.03 - 0.07	0.489
Sex: m	-0.68	-2.12 - -0.77	0.356
Type: papillary carcinoma	-0.43	-2.42 - 1.57	0.675
Stage cat. 2	0.56	-2.42 - 2.05	0.453
Stage cat. 3-4	-0.05	-2.43 - 0.75	0.970
Number of comorbidities cat. 1	-0.84	-2.03 - 1.60	0.298
Number of comorbidities cat. >= 2	-022	-2-03 – 1.60	0.814
Observations	99		

#### 3.4.2 Multiple linear regression model for the IPQ-R questionnaire

In the IPQ-R questionnaire, the inferential analysis shows no significance between the type of carcinoma and the IPQ-R dimensions themselves (**Table 4**). The type of intervention is statistically significant (P = 0.028) in relation to the dimension ‘emotional representations’.

**Table 4:** Comparison by type of carcinoma.

	N	Follicular carcinoma (N=27)	Papillary carcinoma (N=201)	Combined (N=228)	P-value
Identity score	228	1.0/5.0/8.0	2.0/5.0/9.0	2.0/5.0/8.2	0.72
Timeline score	228	7/12/18	11/14/18	10/14/18	0.32
Consequences score	228	20/27/32	21/27/32	21/27/32	0.76
Personal control score	228	14/16/20	15/18/21	14/18/21	0.24
Treatment score	228	46/53/63	50/56/62	50/56/62	0.61
Illness score	228	12/15/20	14/18/20	14/18/20	0.2
Timeline cyclical score	228	4/8/10	7/8/11	6/8/11	0.35
Emotional score	228	11/16/20	12/15/20	12/15/20	0.84
Causes score	228	28/38/46	30/41/47	29/41/47	0.62

**Figure 5:**
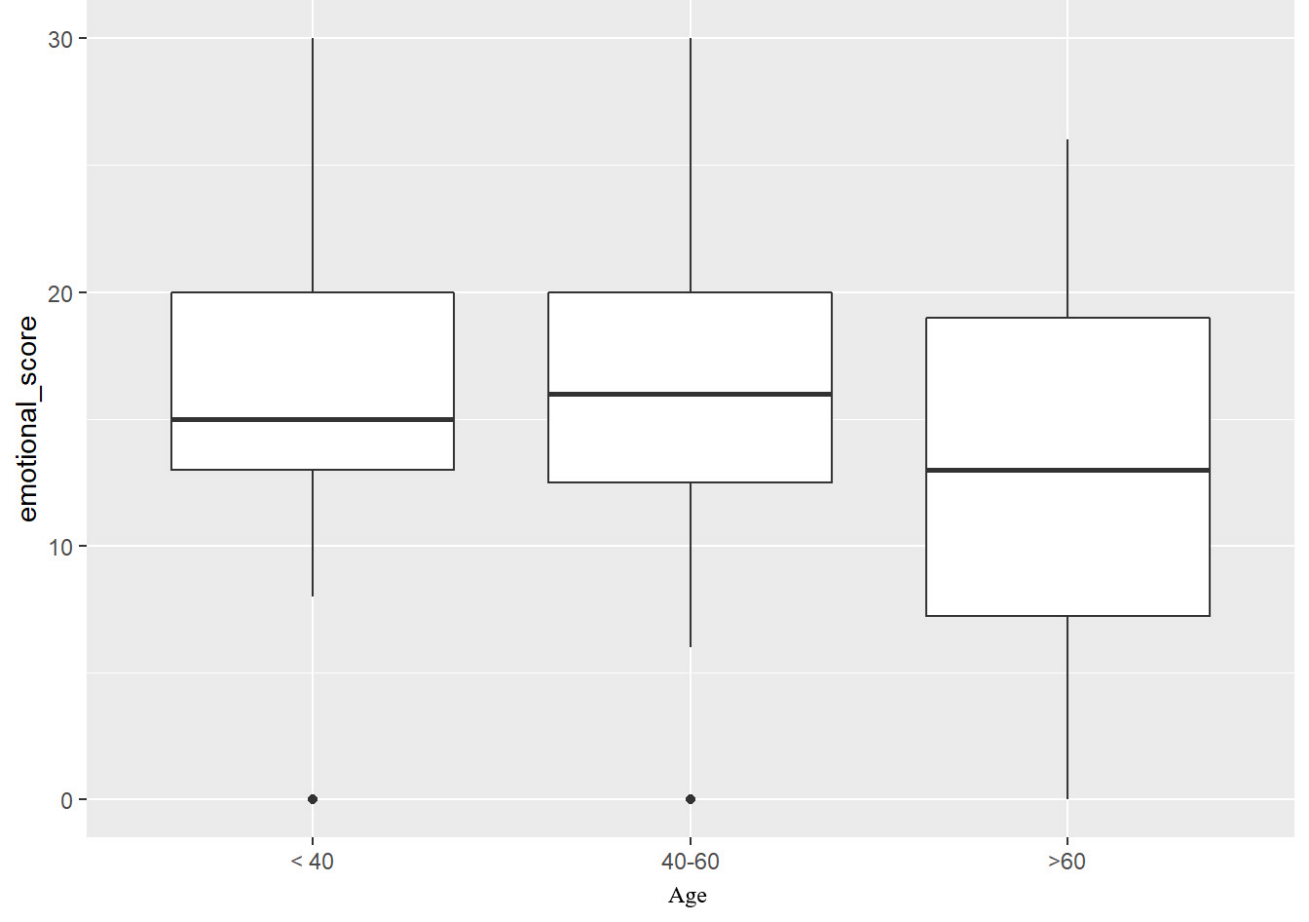
Multiple linear regression model emotional score IPQ-R.

The multiple linear regression model showed the staging of the disease as statistically significant (P = 0.026) in the ‘identity’ section in relation to the mental representation of the disease (**Table 5**). It should be noted that age (P < 0.001), sex (P = 0.026) and number of comorbidities (P = 0.034) are statistically significant with respect to stage. Inferential analysis showed age as statistically significant for the dimension ‘emotional representations’ (P = 0.035) (**Figure 5**).

**Table 5:** Comparison by staging.

	N	1 (N=122)	2 (N=84)	3 (N=9)	4 (N=13)	Combined (N=228)	P-value
Identity score	228	3.0/6.0/9.0	1.0/4.0/7.0	0.0/1.0/10.0	0.0/2.0/4.0	2.0/5.0/8.2	0.026
Timeline score	228	10/14/18	11/14/17	12/20/22	8/14/19	10/14/18	0.73
Consequences score	228	21/27/32	22/27/31	22/33/38	17/25/32	21/27/32	0.75
Personal control score	228	15/18/21	12/19/21	12/16/18	14/17/20	14/18/21	0.35
Treatment score	228	51/56/62	44/56/62	50/58/63	40/55/59	50/56/62	0.82
Illness score	228	16/19/21	13/17/20	16/18/19	14/18/21	14/18/20	0.065
Timeline cyclical score	228	7.0/8.0/10.0	6.8/9.0/12.0	5.0/8.0/9.0	4.0/8.0/14.0	6.0/8.0/11.0	0.75
Emotional score	228	12/16/20	10/14/19	14/16/19	12/14/19	12/15/20	0.14
Causes score	228	32/41/47	15/41/46	0/40/44	26/38/48	29/41/47	0.73

The items ‘personal control’ (P = 0.004), ‘consistency with disease’ (P < 0.001), and ‘causes’ (P = 0.004) also correlated positively with the age variable (**Table 6**).

**Table 6:** Scoring with respect to age.

	< 40 (N=39)	40-60 (N=111)	>60 (N=78)	Combined	P-Value
Identity score	4.5/6.0/8.5	2.0/5.0/9.0	1.0/4.0/6.8	2.0/5.0/8.2	0.094
Timeline score	12.0/14.0/16	10.0/14.0/18.5	8.5/15.0/18.8	10.0/14.0/18	0.99
Consequences score	23/27/32	21/27/33	16/27/32	21/27/32	0.45
Personal control score	16/20/22	15/18/22	9/16/20	14/18/21	0.004
Treatment score	52/58/63	51/56/62	37/54/61	50/56/62	0.068
Illness score	17/20/20	15/19/21	9/16/20	14/18/20	<0.001
Timeline cyclical score	8.0/9.0/10.0	6.5/8.0/11.0	3.2/8.0/12.0	6.0/8.0/11	0.61
Emotional score	13.0/15.0/20	12.5/16.0/20.0	7.2/13.0/19.0	12.0/15.0/20	0.035
Causes score	32/40/47	32/43/48	0/36/43	29/41/47	0.004

#### 3.4.3 Multiple linear regression model for the SF-12 questionnaire

The multiple linear regression analysis for the SF-12 PCS-12 questionnaire revealed that gender, disease staging, and comorbidity status, were associated with variations in PCS-12 scores. In the full sample, males showed significantly higher PCS-12 scores compared to females (Estimate = 2.73, p = 0.037), and individuals with advanced disease stage (stage 3-4) had significantly lower scores (Estimate = -4.12, p = 0.046). Additionally, having zero comorbidities was associated with a substantial increase in PCS-12 scores (Estimate = 5.29, p = 0.002), indicating a positive impact of the absence of comorbidities on physical health status (**Table 7**). When the analysis was restricted to participants with at least one comorbidity, the sample size reduction resulted in the loss of statistical significance for these associations, underscoring the impact of the zero-comorbidity group in driving these effects (**Supplementary Tables S2, S3**).

**Table 7:** Multiple linear regression model for SF-12 PCS-12 questionnaire.

Variables	Estimate	CI	P-Value
Age	0.06	-0.03 – 0.15	0.200
Sex: male	2.73	0.17 – 5.28	0.037
Type: papillary carcinoma	2.72	-0.74 – 6.17	0.123
Stage cat. 2	-0.46	-3.05 – 2.14	0.728
Stage cat. 3-4	-4.12	-8.17 – -0.07	0.046
Number of comorbidities cat. 1	5.29	2.04 – 8.54	0.002
Number of comorbidities cat. >=2	1.19	-2.28 – 4.65	0.501
Observations	179		

Whereas for SF-12 MCS-12 questionnaire, the multiple linear regression model identified age, gender, type of carcinoma, stage of disease and number of comorbidities as not statistically significant in relation to the health status of the patients (**Table 8**).

**Table 8:** Multiple linear regression model for SF-12 MCS-12 questionnaire.

Variables	Estimate	CI	P-Value
Age	-0.02	-0.17 – 0.13	0.779
Sex: male	1.17	-2.96 – 5.30	0.577
Type: papillary carcinoma	-2.28	-7.86 – 3.31	0.422
Stage cat. 2	2.46	-1.73 – 6.66	0.248
Stage cat. 3-4	2.27	-4.27 – 8.82	0.494
Number of comorbidities cat. 1	-1.80	-7.06 – 3.45	0.499
Number of comorbidities cat. >=2	0.71	-4.90 – 6.31	0.803
Observations	179		

## 4 Discussion

### 4.1 Considerations regarding the PGWBI questionnaire

The PGWBI questionnaire was created to provide an index measuring the subjective state or discomfort related to the emotional and affective sphere. The sample presented a median score of 58 in the dimensions ‘anxiety’, ‘depression’, ‘positivity and well-being’, ‘personal control’, ‘general health’ and ‘vitality’, indicating that the patients presented severe distress (17) with regard to the state of psychological well-being. The feelings expressed are states of anxiety, upset, sadness, depression, tension, the presence of physical pain and, sometimes, the feeling of having had little rest during the night. These results are in line with studies by Crevenna R. et al. (2003) (18), by Germano CM. et al. (2016) (19), by Rogers SN. et al. (2017) (20) and by Goswami S. et al. (2018) (21) who confirm the presence of stress, fatigue and insomnia among the individuals they studied. The inferential analysis also allowed to confirm that the state of well-being is influenced by the type of intervention the sample underwent (P = 0.022). Those at risk of a worsening quality of life are those who have undergone total thyroidectomy with lymphadenectomy (Q2: 0) compared to individuals who underwent the operation without lymph node dissection (Q2: 58). It is hypothesized that this is due to the patient’s awareness of neoplastic involvement in other districts, which finds validation in the study by Haraj NE. et al. (2018) (22).

### 4.2 Considerations regarding the IPQ-R questionnaire

#### 4.2.1 Section 1 - identity

It can be inferred from the sample data that the symptoms identified by the patient do not correlate with the current disease. This means that the sample has a good perception and awareness of their disease. Awareness of disease is closely related to staging: specifically, subjects with stage 1 and 2 disease have a good perception of illness, while subjects with stage 3 and stage 4 disease have a lower awareness of illness. This finding is very interesting and in contrast to the study by Husson O. et al. (2013) (23) according to which staging is not a variable correlated with quality of life.

#### 4.2.2 Section 2 - opinions on the illness

This section of the questionnaire provides insight into how the subject perceives his or her illness condition. All seven dimensions were studied in the research. In the dimension ‘timeline’, the sample obtained a median score of 14, indicating a good perception of the course of their disease. Subjects have the perception that their illness will last a reasonable time, not too short, nor will it have a chronic course. This finding is interesting because it contrasts with the study by Rogers SN. et al. (2017) (20) in which fear of recurrence was common in half of the patients with thyroid carcinoma and was considerable/moderate.

The ‘consequences’ dimension, on the other hand, reflects the patient’s perception of the short-term and long-term effects of the disease and the impact on quality of life in physical, psychological and social terms. The sample shows a median of 27, at the upper end of the reference range, an indication that the patients perceive their current illness as potentially dangerous, that it can lead to serious consequences and that it has a major impact on their lives. It should be noted that this negative perception contrasts with other previously reported impressions, according to which the disease was perceived as having a non-chronic course. The fact that the illness is considered potentially dangerous, and fears persist is confirmed in studies by Hedman C. et al. (2017) (24) and Lee KJ. et al. (2016) (25) whose subjects present in a constant state of anxiety, mostly resulting from the risk of recurrence and developing metastatic disease. According to studies by Lubitz CC. et al. (2016) (26) and Germano CM et al. (2016) (19) there is also the patients’ awareness that they must take the hormone replacement for life, which generates a feeling of drug dependency. This can be explained by considering the study by Applewhite MK. et al. (2016) (27) which highlights how health professionals and social media give patients the impression that thyroid cancer is the ‘good cancer’ thereby trivializing the diagnosis. This, as highlighted by the study by Randle RW. et al. (2017) (28), arouses feelings of confusion and frustration. The subjects in the sample studied also perceive how the disease changes the way others see them and affects those close to them (family, friends), confirming the results of the study by Germano CM. et al. (2016) (19) in which patients reported changes in family dynamics and social status after experiencing illness and its treatment.

The ‘personal control’ dimension, with a median of 18, provides a very interesting statistic as it allows to state that attitude and actions may be able, in part, to positively influence the development of one’s clinical picture. Thus, it appears that there is a good perception that one can control the course of the illness through one’s own actions and behaviors. This positive perception is closely correlated with age (P = 0.004). Specifically, individuals over the age of 60 have less perceived control over the disease than individuals between the ages of 40 and 60 and those under the age of 40. This fact explains that older individuals perceive the course of their disease as less dependent on their actions, behaviors and mood. This result is of considerable importance as it allows to reflect on the importance of an intervention aimed at the patient, which is also appropriate to his or her age.

Regarding the dimension ‘treatment control’, the data obtained from the sample show a median score of 56, much higher than the reference range. This result indicates that the patients perceive the treatments performed for the disease as very effective in healing. In particular, subjects display a high degree of confidence in the therapies being implemented and present a strong belief that they can exercise control over the therapy being carried out. This allows to highlight how good patient confidence helps treatment compliance. This relatively positive perception underlines how good communication provided initially helped the patients themselves to become adequately aware of their illness. This thought is in line, in part, with the results of the study by Gamper EM. et al. (2015) (29) according to which the patients, aware that they had the ‘good cancer’, felt feelings of reassurance and high confidence towards the treatments they were undergoing and towards the course of the disease.

In the dimension ‘consistency’, the sample obtained a median score of 18, within the reference ranges. The patients’ perception and understanding of their illness is good and they are aware of what they have and what they feel. It is hypothesized that this perception is always derived from adequate information given to the patients as soon as they are taken into care, i.e. when the nature of the disease and what changes in terms of physical and mental health the removal of the thyroid gland entails are explained to them. Again, age significantly (P < 0.001) influences this perception. In particular, patients over 60 years of age understand their disease less clearly (Q2: 16) compared to patients under 40 years of age (Q2: 20).

The dimension ‘emotional representations’ achieved a median score of 15, within the reference ranges. The sample, with respect to disease, has no prevalence of positive or negative emotions. Sometimes feelings of serenity emerge, sometimes emotions of anger and concern towards the illness. In line with studies by Haraj NE. et al. (2018) (22), by Lee KJ. et al. (2016) (25), by Rogers SN. et al. (2017) (20) and by Hedman C. et al. (2017) (24) one hypothesis for the appearance of negative feelings may be the fear of recurrence or the appearance of other tumors. The inferential analysis also revealed that the perception of the sample changes in relation to the variables listed below. The type of intervention is statistically significant (P = 0.028) in relation to the emotional representations of the patients. Those who have undergone total thyroidectomy have a higher prevalence of ‘negative’ emotions than those who have also undergone lymph node dissection. Age was again statistically significant (P = 0.0035). In particular, individuals over 60 years of age have a higher prevalence of positive emotions, are less angry and less worried than individuals under 60 years of age. This finding is in line with studies by Aschebrook-Kilfoy B. et al. (2015) (30), by Germano CM. et al. (2016) (19), by Goldfarb M.et al. (2015) (31) and by Duan H. et al. (2015) (32) stating that older individuals present a better quality of life than younger individuals, who present an impaired quality of life, experience feelings of anxiety and depression mainly because they are afraid to return to work for fear of being judged or treated differently. According to the study by Abdul-Sater L. et al. (2011) (33) this anxiety is probably also influenced by the young person’s past experience, personality and coping style. The relationship between age and emotional representations of cancer disease is certainly influenced by several factors: individuals of different ages react emotionally in distinct ways to a cancer diagnosis, mainly due to their life experiences, expectations and personal resources. There is no correlation between age and perception of illness but a significant correlation between level of education and perception of disease exists (3). This suggests how this is an area to be investigated further in the future in order to then be able to offer ‘tailor-made’ treatment.

The last dimension is ‘cyclicality’. The patients obtained a median score of 8, a value close to the theoretical minimum, an indicator that the sample does not perceive the illness as cyclical, but that their present symptoms are constant over time and do not vary in relation to the day.

To contextualize the results within the existing literature and to investigate the psychological and social dynamics that may influence the distress and psychological well-being of patients with regard to elderly patients, they present a different, sometimes more serene view of the illness. This may be explained by having already faced other difficulties in life. Some report greater frailty and fear of death in the course of their treatment, and this naturally influences their emotions. Furthermore, it should be analyzed how invasive procedures such as total thyroidectomy with lymphadenectomy can impact psychological well-being. It will help clarify the practical implications for pain management and psychological support. Delving into these aspects in future research may help to suggest targeted interventions to improve patients’ quality of life.

Patients over 60 years of age, despite the prevalence of ‘positive’ emotions, perceive that they have less control over the disease and consider it to be less dependent on their actions, attitudes and moods. Moreover, they have a less clear understanding of its nature. This can be explained in several ways. Perhaps many may be more focused on the management of other health issues and less on cancer itself. Their previous life experiences might also act as a ‘protective’ factor, in the knowledge instead of their own emotions. In the under-60s, on the other hand, the course of treatment, with surgery and possible long-term consequences on the quality of life may be a worrying factor, thus increasing the negative perception of the disease.

#### 4.2.3 Section 3 - causes of the disease

The third section of the IPQ-R questionnaire, ‘causes of the illness’, assesses the patients’ opinions on the factors that may have caused the disease. The multiple linear regression model shows that age is a statistically significant correlated variable (P = 0.004), which allows to hypothesize that it may be a factor influencing the motivations that patients attribute to the illness. This is also found in the article by Trovato G.M. et al. (2006) (12).

### 4.3 Considerations regarding the SF-12 questionnaire

The questionnaire was developed to measure the health status of a group of people through two synthetic indices, the PCS-12 and the MCS-12.

#### 4.3.1 PCS-12 index

This sample presents a median score of 51 out of a theoretical maximum of 100 points in relation to the dimensions ‘physical activity’, ‘role and physical health’, ‘physical pain’ and ‘general health’. This score, which is intermediate in relation to the reference scale, indicates how, over the past four weeks, patients have underperformed. This finding is confirmed by the article by Crevenna R. et al. (2003) (18) whose patients show significant deficits in HRQOL domains such as ‘vitality’, ‘role physical’, ‘mental health’, ‘role emotional’ and ‘social functioning’. These dimensions are significantly impaired during the first year after diagnosis. In this study’s sample, patients experienced partial limitations in self-care (performing personal hygiene, taking care of one’s external appearance) and presented physical pain and fatigue accompanied by low energy that partially hindered activities of daily living. This finding is confirmed by the article by McIntyre C. et al. (2018) (34) in which patients confirmed a limitation in performing activities of daily living. This study also makes it possible to state that even in tasks of moderate physical exertion (moving a table, vacuuming, going for a bike ride), the sample showed partial limitation, even though their general health was good most of the time, this being due to the fact that the effect on quality of life is mainly related to the emotional and social impacts of the treatment (26). The latter is a very interesting result and not in line with the study by Rogers SN. et al. (2017) (20), whose article shows that the most affected domain in the sample was overall health status followed by cognitive and emotional function.

The statistically significant differences observed between genders, disease stage (stage 3-4 vs stage 1), and number of comorbidities (1 vs 0) can be considered in clinical practice to orient the focus on patients with higher risk of unsatisfactory performance.

Looking at **Figure 2** (Density Plot PCS-12 score), it can also be stated that the variability in the distribution below the median score of 51 emphasises that patients in that category present significant physical pain and frequent fatigue.

#### 4.3.2 MCS-12 index

In relation to the MCS-12 index of the SF-12 questionnaire, patients presented a median score of 57 out of a theoretical maximum of 100 points in terms of ‘vitality’, ‘social activities’, ‘emotional state’ and ‘mental health’. It can be deduced from these data that the sample in this study sometimes experiences difficulties in concentrating at work or in other activities, but most of the time they experience serenity, sometimes feelings of sadness, which partly interfere with social and family activities and outings with friends. The inferential analysisdid not reveal significance between the variables ‘age’, ‘sex’, ‘type of carcinoma’, ‘staging’ and ‘number of comorbidities’, not allowing to identify patients at risk of poorer mental health. In conclusion, it should be emphasised one last very interesting aspect in relation to the multiple linear regression model. In all questionnaires, inferential analysis showed that female sex and the presence of comorbidities were not predisposing variables for a worsening quality of life. This is an innovative finding as it departs from the studies of Aschebrook-Kilfoy B. et al. (2015) (30), by Crevenna R.et al. (2003) (18), by Husson O. et al. (2013) (23), by Goldfarb M. et al. (2016) (31), by Jung MS. et al. (2017) (35) and by Pak K. et al. (2018) (36) in which two concepts are affirmed. Firstly, their studies state that comorbid conditions may influence the presentation and recognition of symptoms and thus lead to a worsening of quality of life; secondly, they claim that the female sex is seen as more vulnerable, with greater symptoms of fatigue and a worsening of cognitive functions.

### 4.4 Limits of the study

The results of the study must be interpreted with caution with reference to the marked imbalance between the group of patients undergoing total thyroidectomy with lymph node dissection and the group without: 5 vs 223. Also, with reference to the data analysis conducted with regard to carcinoma staging, the distribution of patients in the different classifications is decidedly unbalanced (third and fourth stage 9 and 13 patients respectively). The small number of patients with follicular carcinoma, 27, compared to patients with papillary carcinoma, 201, must also be considered when interpreting the study results. A further limitation is the fact that the questionnaires were administered on the first day of admission to the protected section, giving the opportunity to complete them over the following three days. Participants who completed questionnaires may have done so at different stages of treatment, which may have influenced the measurement of quality of life on those days. The lack of data on the time elapsed between total thyroidectomy surgery with/without lymphadenectomy and admission to the protected section is also a limitation. According to studies by Crevenna et al. (2003) (18) and by Haraj NE. et al. (2018) (22) the domains of ‘mental health’, ‘role emotional’, ‘role physical’, and ‘social functioning and vitality’ are significantly impaired during the first year after diagnosis, and then improve. Another limitation is that the type of comorbidity in the individuals studied was not considered. As the concomitant diseases were extremely scattered, this variable was not considered in the statistical analysis. Another limitation of the study is the lack of detailed information on the types of comorbidities. As a result, the analysis was based on the number of comorbidities and the presence or absence of comorbid conditions, rather than grouping comorbidities by type. This approach allowed to capture the overall comorbidity burden but may limit the ability to draw conclusions about the impact of specific types of comorbidities on quality of life.

## 5 Conclusions

Concerning the state of psychological well-being, moderate/severe distress was shown by the sample and the feelings expressed were anxiety, upset, sadness, depression and tension; all emotions that, to some extent, interfered with social and family activities. Regarding the perception of health and illness, very interesting results emerged. The subjects presented a good perception and awareness of their disease, they are convinced that their disease will last for an appropriate length of time and are aware that their attitude and actions may be able, in part, to positively influence the development of their clinical picture. There is also a high level of confidence in the treatments carried out for the disease, and the therapies are considered effective for their recovery. Despite these positive perceptions, emotions of anger and concern were sometimes registered towards the illness, which was seen by the patients as potentially dangerous and having a major impact on their lives.

In relation to the state of physical well-being, the sample presented partial limitations in self-care (performing personal hygiene, taking care of one’s external appearance) and in moderately physically demanding tasks (moving a table, vacuuming, riding a bicycle). They presented muscular pain, tiredness and sometimes the feeling of having had little rest during the night. Only a portion of the sample presented significant pain and constant fatigue. These symptoms, characterised by cyclicality, were accompanied by low energy that partially hindered activities of daily living.

With respect to the second objective, categories of individuals at risk and into whom care resources could be invested were identified:

- Patients with stage 3 and 4 thyroid carcinomas as they presented less disease awareness;
- Patients over 60 years of age as, despite the prevalence of ‘positive’ emotions, they have less perceived control over the disease, perceive its course as less dependent on their actions, conduct and mood, and understand its nature less clearly;
- Subjects under 60 years of age as they have a prevalence of ‘negative’ emotions and worries;
- Subjects who have had total thyroidectomy surgery without lymph node dissection as they report a prevalence of ‘negative’ emotions.

### 5.1 Implications for practice

The sample aged less than 60 years and having undergone total thyroidectomy surgery without lymphadenectomy has the perception that the illness is potentially serious and presents concerns. This may be explained by the fear of developing a metastasis or recurrence (20, 24, 25). Moreover, routine surveillance and required examinations are a reminder of the disease and this creates a constant state of alertness (19). According to the study by Duan H. et al. (2015) (32), health professionals can contribute by explaining to patients, from the moment he or she is taken on as a patient, the nature of his or her disease, and by reiterating to the patient that the frequency of laboratory tests and checks that are scheduled and will be carried out do not necessarily correlate with the prognosis. This improves information, reduces stress and increases the degree of adaptation of the patients themselves.

With patients over 60 years of age and stage 3 and 4 disease, as mentioned in the study by Liu T. et al. (2019) (37), action can be taken, in order to increase awareness, by informing patients even before the intervention about the hypothyroid condition that could occur before the appropriate dose of replacement hormone is found and informing subjects that this could cause an increase in physical fatigue as well as a change in mood (37, 38). In addition to these interventions, the presence of a moderate/severe state of distress among patients in relation to psychological well-being, with the presence of feelings of anxiety, upset, sadness, depression and tension, emphasises the need to activate psycho-oncological support throughout the entire course of treatment, from the first visit to the follow-up phase.

In relation to physical health, regarding the moderate/severe fatigue found in the sample, strategies to improve symptoms are proposed in the literature. For example, Crevenna R. et al. (2003) (18) argues that early exercise, particularly endurance and strength training performed in a group, increases vitality and improves all dimensions of quality of life, including regaining one’s social role (family, friends, work). Kim K. et al. (2018) (39) found home exercise to be equally effective in reducing tiredness, fatigue, stress and anxiety, leading to increased vitality and improved immune function. It follows that an exercise programme aimed especially at individuals who have undergone a total thyroidectomy and are younger than 60 years of age could be useful: performing aerobic exercises at an average of 120 minutes per week, resistance exercises twice a week on average, and flexibility exercises 10 minutes before and 10 minutes after resistance exercise. It can also be recommended to establish a physical activity programme at home as part of a long-term strategy (39).

### 5.2 Implications for research

The study is limited by its cross-sectional design. A longitudinal study through further administration of the validated questionnaires PGWBI, IPQ-R and SF-12 would make it possible to assess whether and how quality of life and perceptions of health and illness have changed over time. Future research should also focus on how invasive procedures such as total thyroidectomy with lymphadenectomy can impact psychological well-being to clarify the practical implications for pain management and psychological support suggesting targeted interventions to improve patients’ quality of life. Delving into the mechanisms underlying the associations between age and perception of the disease could provide important insights into how different age groups cope with the disease. Furthermore, the inclusion of qualitative data in future research could greatly enrich the understanding of patients’ experiences. Quantitative data offer objective and statistical measurements, qualitative information can reveal emotional parts of patients. This integrated approach would allow to delineate nuances about the emotional individuality of patients, thus improving the interpretation of results and revealing a more comprehensive view of the emotional impact of illness on patients. Ultimately, this would have an impact on clinical practice, allowing the implementation of more targeted interventions that are sensitive to patients’ needs, increasing the overall value of the present study.

### 5.3 Key findings

Key findings and their clinical implications are summarised in **Table 9**.

**Table 9:** Key findings and their clinical implications.

CONCLUSIONS	IMPLICATIONS FOR PRACTICE
Psychological well-being: moderate/severe distress and the feelings expressed were anxiety, upset, sadness and depression and these emotions interfered with social and family activities. Emotions of anger and concern were registered towards the illness. People presented a good perception and awareness of their disease and they had high level of confidence in the treatment. Physical well-being: the sample presented partial limitations in self-care and in demanding tasks like moving a table or vacuuming. They presented muscular pain, tiredness and sometimes the feeling of having had little rest during the night. These symptoms are characterized by cyclicality and are accompanied by low energy. In order to improve and personalise care, certain categories of patients at risk of a lower quality of life have been identified: • Patients with stage 3 and 4 thyroid carcinomas: they show reduced awareness of the disease. • Patients over 60 years of age: despite the prevalence of ‘positive’ emotions, these patients perceive that they have less control over the disease and consider it to be less dependent on their actions, attitudes and state of mind. They also have a less clear understanding of its nature. • Patients under 60 years of age: they have a prevalence of ‘negative’ emotions and worries. • Patients undergoing total thyroidectomy without lymph node dissection: report a prevalence of ‘negative’ emotions.	In patients under 60 who have had a total thyroidectomy without lymphadenectomy, the illness feels severe, and routine follow-ups serve as constant reminders, leading to heightened vigilance. Duan H. et al. (2015) suggest that healthcare providers can support these patients by clearly explaining the nature of their disease, which enhances understanding, reduces stress, and helps patients adapt better to their condition. For patients over 60 with stage 3 or 4 disease, it is important to inform them before treatment that they may experience hypothyroidism until the correct dose of hormone replacement is determined, which could lead to increased fatigue and mood changes. Additionally, due to moderate to severe psychological distress—characterized by anxiety, sadness, depression, and tension—psycho-oncological support should be provided throughout their treatment. Exercise, especially endurance and strength training done in groups, boosts vitality and enhances overall quality of life. Consequently, incorporating an exercise program can be beneficial. Additionally, setting up a home-based physical activity routine can serve as an effective long-term strategy for sustaining these benefits.

## Data Availability

The raw data supporting the conclusions of this article are available at https://doi.org/10.5281/zenodo.14389731.
