# Supplementary material for: Perception of health and illness and quality of life after total thyroidectomy for differentiated thyroid carcinoma: the PERSAM study
Source: Front Endocrinol (Lausanne). 2024 Dec 26;15:1472448. doi: 10.3389/fendo.2024.1472448 (PMC11714454; doi:10.3389/fendo.2024.1472448)
Supplement: Supplementary file 1 [file Table1.docx]

Supplementary material

Table S1. Multiple linear regression model for PGWBI questionnaire in participants without comorbidities

| **Variables** | **Estimate** | **CI** | **P-Value** |
| --- | --- | --- | --- |
| Age | -0.00 | -0.08 - 0.08 | 0.973 |
| Sex: m | -0.36 | -2.54 - 1.82 | 0.744 |
| Type: papillary carcinoma | 0.38 | -2.96 - 3.72 | 0.823 |
| Stage cat. 2 | 0.04 | -2.25 - 2.33 | 0.974 |
| Stage cat. 3-4 | 0.63 | -2.82 - 4.07 | 0.719 |
| Number of comorbidities cat. 1 | -0.88 | -2.95 - 1.20 | 0.404 |
| Observations | 99 |  |  |

Table S2. Multiple linear regression model for SF-12 PCS-12 questionnaire

| **Variables** | **Estimate** | **CI** | **P-Value** |
| --- | --- | --- | --- |
| Age | 0.13 | -0.04 - 0.31 | 0.137 |
| Sex: male | 4.38 | -0.46– 9.22 | 0.075 |
| Type: papillary carcinoma | 3.47 | -3.26 - 10.21 | 0.307 |
| Stage cat. 2 | -1.93 | -6.94– 3.08 | 0-445 |
| Stage cat. 3-4 | -5.78 | -12.89– 1.32 | 0.109 |
| Number of comorbidities cat. 1 | 1.31 | -3.38– 6.00 | 0.579 |
| Observations | 78 |  |  |

Table S3. Multiple linear regression model for SF-12 MCS-12 questionnaire

| **Variables** | **Estimate** | **CI** | **P-Value** |
| --- | --- | --- | --- |
| Age | -0.09 | -0.30– 0.12 | 0.401 |
| Sex: male | -1.46 | -7.34– 4.42 | 0.622 |
| Type: papillary carcinoma | -2.06 | -10.25– 6.12 | 0.617 |
| Stage cat. 2 | 2.89 | -3.20– 8.99 | 0.347 |
| Stage cat. 3-4 | 0.58 | -8.06– 9.22 | 0.894 |
| Number of comorbidities cat. 1 | 0.24 | -5.46– 5.94 | 0.933 |
| Observations | 78 |  |  |
